# Harvesting Risk: An Ecologic Study of Agricultural Practices and Patterns and Melanoma Incidence in Pennsylvania

**DOI:** 10.1200/CCI-25-00160

**Published:** 2025-11-14

**Authors:** Benjamin J. Marks, Jiangang Liao, Charlene Lam, Camille Moeckel, Eugene J. Lengerich

**Affiliations:** ^1^ Penn State College of Medicine, Hershey, PA; ^2^ Department of Public Health Sciences, Penn State College of Medicine, Hershey, PA; ^3^ Penn State Cancer Institute, Penn State Health, Hershey, PA; ^4^ Department of Dermatology, Penn State Health, Hershey, PA; ^5^ Department of Family and Community Medicine, Penn State College of Medicine, Hershey, PA

## Abstract

**PURPOSE:**

To examine the geospatial distribution of melanoma incidence in Pennsylvania (PA), quantify its association with agriculture practices and patterns, and consider its relevance for cancer control.

**METHODS:**

The study used an ecologic design with county-level PA data on the 2017-2021 incidence of invasive melanoma among adults 50 years and older, as well as agricultural patterns and practices, ultraviolet radiation (UVR), and demographics/socioeconomics. Spatial clustering was examined using local indicators of spatial association and Getis-Ord Gi*. Separate adjacency-weighted Conway-Maxwell-Poisson models, adjusted for UVR and social vulnerability, quantified the association between melanoma and (1) cultivated and pasture/hay acreage and (2) herbicide-, insecticide-, fungicide-, and manure-treated acreage.

**RESULTS:**

Melanoma incidence was 57.1% greater in a 15-county cluster (*P* < .05) in South Central PA; eight counties were designated as metropolitan. Compared with noncluster counties, cluster counties had significantly more cultivated land (mean 19.8% *v* 6.9%, *P* < .001) and herbicide-treated land (16.8% *v* 6.5%, *P* < .001). In adjusted models, a 10% increase in cultivated land and a 9% increase in herbicide-treated acreage each independently corresponded to a 14% increase in incidence.

**CONCLUSION:**

Melanoma incidence clustered in South Central PA, an area with substantial agricultural industry. However, a majority of counties in the cluster were designated as metropolitan, challenging the concept that agriculture is primarily an industry of counties designated as nonmetropolitan (rural). Agricultural practices and patterns were associated with incidence, suggesting that cancer control adopt an integrated One Health approach to concurrently address occupational, environmental, and behavioral risks. The cluster was entirely within the 28-county catchment area of the Penn State Cancer Institute, demonstrating the utility of geospatial data and analysis for cancer control by a cancer center.

## INTRODUCTION

### Melanoma Incidence and Risks

The incidence of melanoma has tripled in the United States since 1975.^1^ While therapy has improved survival in recent years, an estimated 8,400 people will die of melanoma in 2025.^2^

**CONTEXT**

**Key Objective**
Are county-level agricultural practices and patterns associated with melanoma incidence among adults 50 years and older in Pennsylvania (PA), after accounting for ultraviolet radiation (UVR) and social vulnerability, and is there high-incidence geospatial clustering?
**Knowledge Generated**
We identified a 15-county high-incidence melanoma cluster in South Central PA. In models adjusted for UVR and social vulnerability, counties with a greater proportion of cultivated land or herbicide-treated acreage had higher melanoma incidence.
**Relevance *(J.L. Warner)***
This study adds to a growing literature that highlights the importance of environmental considerations in cancer incidence and etiology.*
*Relevance section written by *JCO CCI* Editor-in-Chief Jeremy L. Warner, MD, MS, FAMIA, FASCO.

Melanoma has multiple risk factors including male sex, age, and rural residence, along with family history, previous history of melanoma, genetic background, numbers of melanocytic nevi, and skin color.^3^ Critically, ultraviolet radiation (UVR) is the leading environmental risk factor for melanoma.^4-6^ While persons in outdoor occupations might have substantial ambient UVR exposure,^7,8^ studies of their melanoma risk have shown mixed results,^9-11^ possibly reflecting misclassification of occupation, exposure, and outcomes.^10^

Results from studies assessing melanoma risk among agricultural workers, who have substantial ambient UVR exposure, remain mixed. Several studies report no association,^12-14^ whereas others, including the International Consortium of Agricultural Cohort Studies and its US-based Marshfield Epidemiologic Study Area Farm subcohort, found elevated risk.^15-18^ Importantly, subgroup analyses found variation in risk for farm managers, crop workers, and livestock handlers.^19-21^ These differences may reflect heterogeneity in specific exposures, such as ambient UVR and agrochemicals, including pesticides.^8,17,22^ Indeed, pesticide use, in both occupational^23^ and residential settings, has been implicated in melanoma risk.^24^ Mechanistically, pesticides, which include herbicides, insecticides, and fungicides, may contribute to melanoma pathogenesis by enhancing photosensitivity, suppressing immune function, disrupting hormonal and cell cycle regulation, and inducing oxidative stress and DNA damage.^25-27^

### Pennsylvania Agriculture and Melanoma Risk

Pennsylvania (PA) ranks 11th nationally in agricultural workforce size, with over 600,000 workers on 49,000 farms spanning 7 million acres.^28,29^ Although PA is largely rural, agriculture is concentrated in the south central and southeastern regions. The state's most predominant crops—grasses and hay (34%), corn (28%), and oilseeds (15%)^29^—are among those treated with pesticides.^30^ Population-level estimates suggest that workers involved in corn, soybean, and grain production experience elevated exposure to potentially carcinogenic pesticides.^30^ These risks may be heightened in PA, where exposure may be prolonged because agricultural workers in the state average 55 years of age and have one of the highest job tenures in the United States at 22 years.^29^

### Hypothesis and Study Objectives

**FIG 1.**
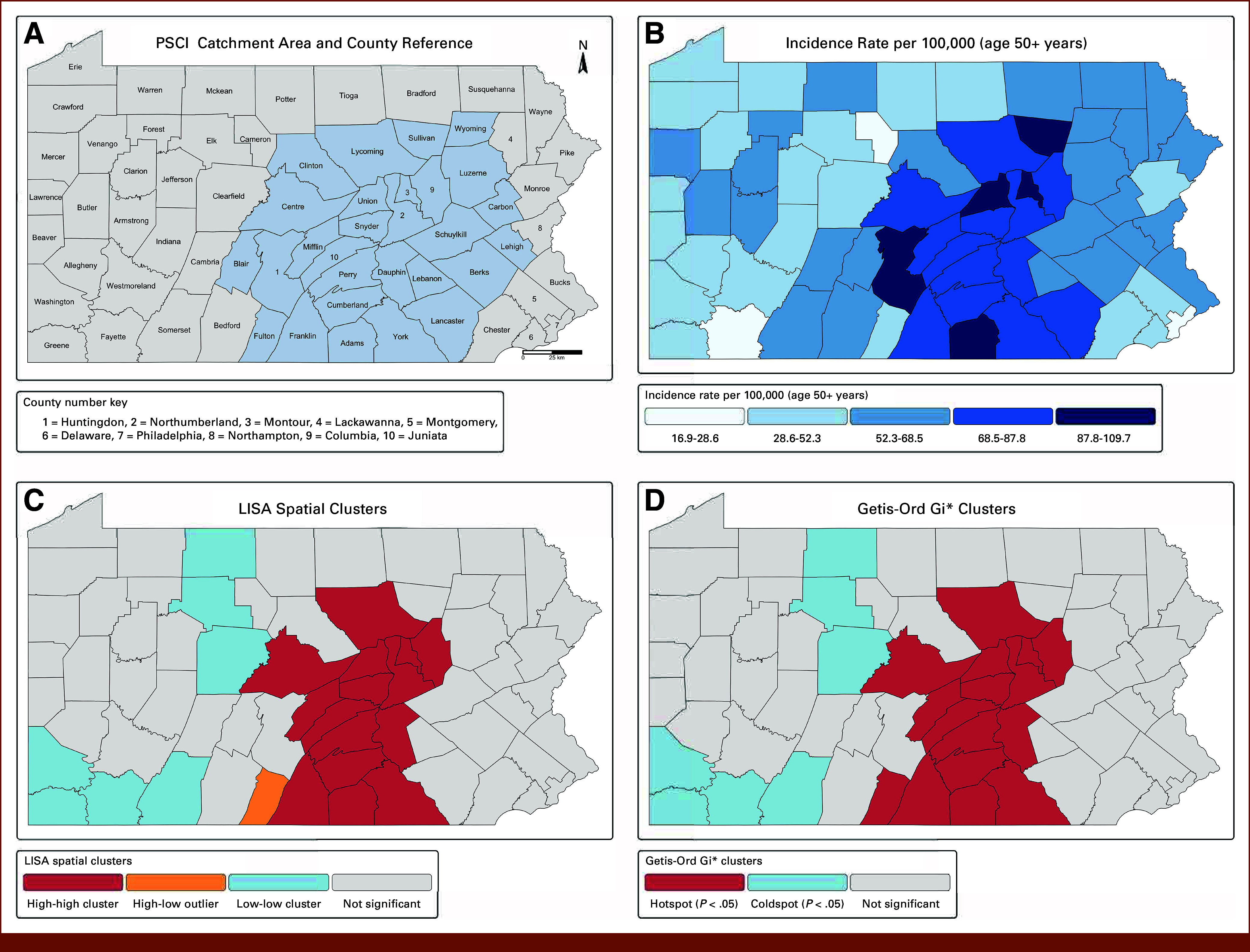
Spatial distribution and clustering of melanoma incidence among adults 50 years and older in PA, 2017-2021. County-level maps of melanoma incidence spatial clustering across PA using four panels: (A) PSCI catchment area and county reference map, with PSCI catchment counties shaded in blue and numbered counties for label adjustments; (B) melanoma incidence rates (cases per 100,000 among adults 50 years and older); (C) LISA identifying high-high clusters (hotspots), low-low clusters (cold spots), and spatial outliers based on county incidence rates; and (D) Getis-Ord Gi* analysis detecting statistically significant hotspots and cold spots using a six-nearest neighbor (KNN) spatial weight matrix. Spatial significance was defined as *P* < .05. Red shading denotes statistically significant high-incidence clusters; blue shading denotes statistically significant low-incidence clusters; and yellow indicates spatial outliers. LISA, local indicators of spatial association; PA, Pennsylvania; PSCI, Penn State Cancer Institute.

Our working hypothesis was that agricultural practices and patterns increase risk for melanoma, even while accounting for ambient UVR and sociodemographic factors. The results of the current study would be particularly relevant for PA because of its large agricultural industry and rural area. Thus, the objectives of this study were to (1) identify geospatial patterns of melanoma and agricultural practices and patterns in PA, (2) assess and quantify the association of agricultural practices and patterns with melanoma incidence, and (3) estimate the proportion of melanoma incidence that may be attributable to agricultural practices and patterns. If our hypothesis is supported, cancer centers with a catchment area that include substantial agriculture industry and rural populations, such as the Penn State Cancer Institute (PSCI; Fig 1A), can initiate studies to reduce melanoma morbidity and mortality.^31^ Indeed, PSCI has already demonstrated limited usage of UVR protective measures among agricultural workers.^32^

## METHODS

### Study Design and Data

We conducted an ecologic study with county-level, cross-sectional secondary data for PA. Key variables included (1) melanoma incidence, (2) agricultural land use, (3) agrochemical-treated acreage, (4) ambient UVR, and (5) social vulnerability.

The primary outcome was county-level incidence of invasive melanoma (T1 and above)^33^ diagnosed between 2017 and 2021 among adults 50 years and older. This age group accounted for 85% of all melanoma cases.^34^ Five-year age-specific incidence rates were calculated using county population estimates. Case and population data were provided by the PA Cancer Registry, which has received the highest certification level for completeness and accuracy since 2014.^35^

Agricultural land-use data were obtained from the National Land Cover Database,^36^ which used satellite imagery to estimate the percentage of county land area for agricultural use, classified into Pasture/Hay (livestock grazing and perennial hay production) and Cultivated Crops (row crops, perennial crops, and tilled land).^37^

Agrochemically treated acreage was estimated using data from the US Department of Agriculture's Census of Agriculture (2012, 2017, and 2022), obtained via the National Agricultural Statistics Service Quick Stats database.^38^ Separate variables for herbicide-, insecticide-, fungicide-, and manure-treated acreage were obtained. We averaged data across all three censuses or two censuses when a single data point was missing. We imputed zero where data were suppressed, which was insecticide use in Pike County and fungicide use in Cameron, Forest, Philadelphia, and Pike Counties. We calculated each agrochemical-treated acreage as a percentage of total county land area.

Ambient UVR data were derived from satellite and ground sensors and obtained from the GIS and Science for Cancer Control platform.^39^ This measure of long-term average daily UVR exposure, reported in Watt-hours per square meter (Wh/m^2^), has remained stable from 1975 through 2005, supporting its use in this study as average exposure.^40^

We assessed county sociodemographics using the 2022 Social Vulnerability Index (SVI).^41^ Our primary measure was the overall SVI percentile rankings (RPL_THEMES), which summarizes four domains: socioeconomic status, household composition/disability, racial/ethnic minority status, and housing/transportation. Percentile ranking values range from 0 to 1, with higher values indicating greater social vulnerability. To explore potential effects of the minority population, we included SVI Theme-3 (SVI-T3; ie, percentage of the population that was Hispanic or non-White) as a separate covariate in sensitivity analyses.

### Research Protection

This project was determined to not constitute human participant research by the Office for Research Protections, The Pennsylvania State University (STUDY00026481).

### Statistical Analysis

Descriptive statistics were calculated for all variables. Geospatial clustering of melanoma incidence was evaluated using two methods: local indicators of spatial association (LISA) and the Getis-Ord Gi* statistic. LISA, based on local Moran's I and a 6-nearest neighbors (6-NN) spatial weights matrix, identified counties with significantly high or low incidence relative to their neighbors, classifying them as high-high clusters (hotspots), low-low clusters (cold spots), or spatial outliers.^42^ The Getis-Ord Gi* statistic independently assessed clustering using weighted local averages (6-NN).^43^ Geospatial significance was defined at *P* < .05, and maps were generated using the *tmap* package in R.^44^

To assess associations with melanoma incidence, we fit trivariate (agro-variable + UVR + SVI) and multivariable Poisson regression models in two exposure tracks: (1) land-use variables and (2) agrochemical variables. All models included UVR and SVI as covariates and used the log-transformed population 50 years and older as the population offset. Our use of the ≥50 population minimized dilution by low-risk younger groups and improved model interpretability.

Continuous variables were standardized using z-scores before modeling. Multicollinearity was assessed using variance inflation factors (VIFs), and covariates exceeding a VIF of 10 were excluded. Because of observed overdispersion, underdispersion, and geospatial autocorrelation, all final models were refit using adjacency-weighted Conway-Maxwell Poisson regression via the *spaMM* package.^45-47^ These models incorporated county-level random effects estimated using penalized maximum likelihood and used a queen-contiguity spatial weights matrix. Model performance was evaluated using log-likelihood, marginal Akaike information criterion (mAIC), conditional AIC (cAIC), and pseudo-R^2^ values. Moran's I confirmed no significant residual spatial autocorrelation. The Data Supplement (Tables S1-S15) shows extended regression results, sensitivity analyses, multicollinearity diagnostics, and Poisson/quasi-Poisson model outputs. All analyses were conducted in R (v4.4.2) using *spaMM*, *spdep*, *sf*, *tmap*, and *tidyverse* packages.^44,47-51^

## RESULTS

### Melanoma Incidence and Geospatial Clustering

The average melanoma incidence for PA counties was 60.4 cases per 100,000 adults 50 years and older, with rates ranging from 16.9 to 109.7 per 100,000 (Fig 1B; Data Supplement, Table S1A).

Geospatial analyses identified nearly identical clustering patterns (Figs 1C and 1D). LISA detected a statistically significant (*P* < .05) high-high cluster, meaning counties with elevated incidence adjacent to similarly high-incidence counties. The cluster consisted of 15 contiguous counties, hereafter referred to as the south central cluster: Adams, Center, Columbia, Cumberland, Dauphin, Franklin, Juniata, Lycoming, Mifflin, Montour, Northumberland, Perry, Snyder, Union, and York. Low-low clusters were detected in northern and southwestern PA. Getis-Ord Gi* corroborated these findings, identifying the same hotspot (plus Fulton County; *P* < .05) and identical cold spots. The mean melanoma incidence in the south central cluster was 84.2 per 100,000, 1.57 times higher than that in the other PA counties (53.6 per 100,000; Data Supplement, Table S1A).

### Geospatial Distribution of Explanatory Factors

**FIG 2.**
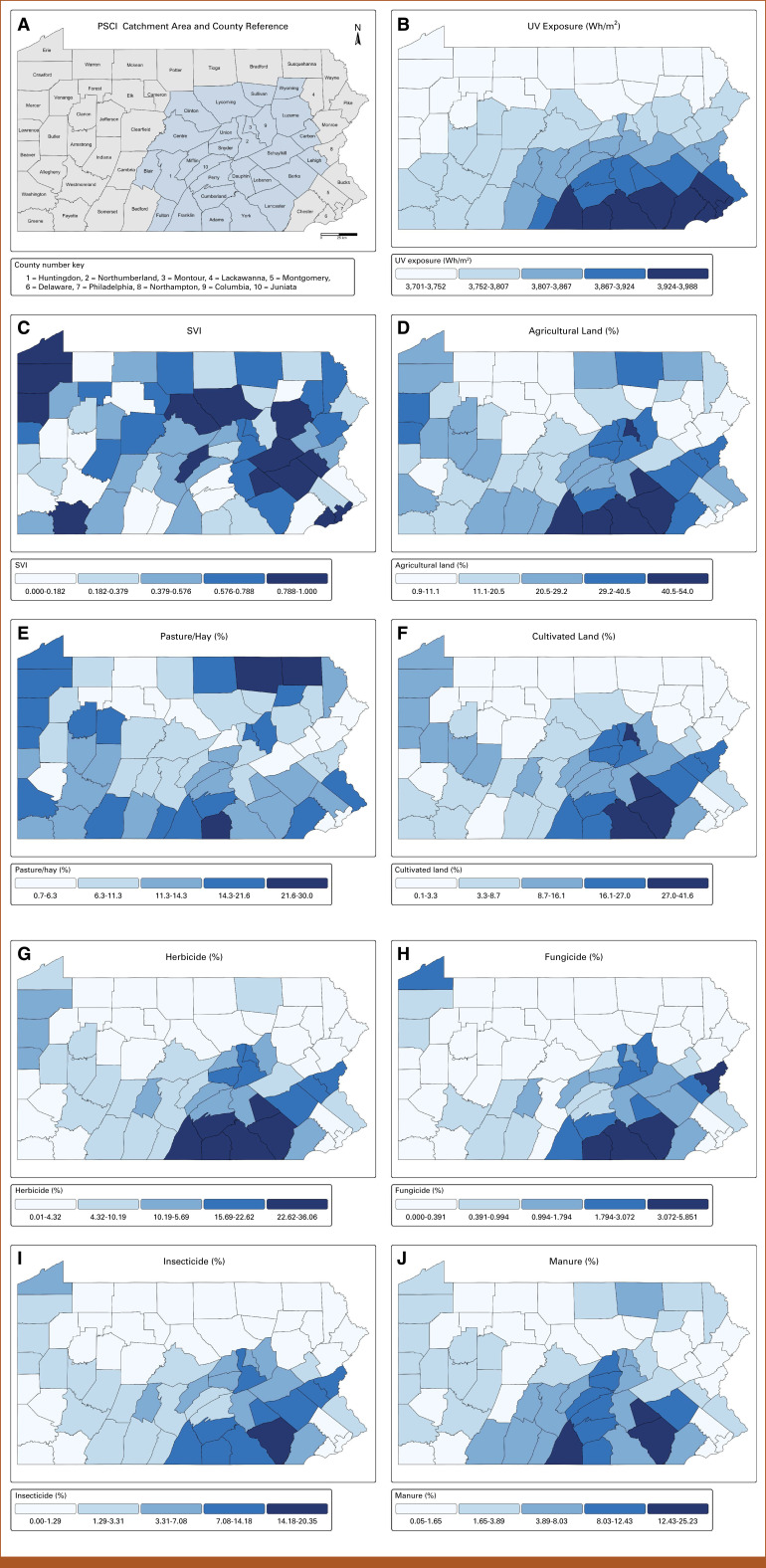
Geographic distribution of environmental, agricultural, and sociodemographic factors across Pennsylvania counties. County-level maps displaying the following variables: (A) PSCI catchment area and county reference map, with PSCI catchment counties shaded in blue and numbered counties for label adjustments; (B) UVR exposure in watt-hours per square meter (Wh/m^2^); and (C) SVI percentile. Land use and agrochemical exposures as percentages of total county land area, including (D) total agricultural land, (E) pasture/hay land, (F) cultivated land, (G) herbicide-treated acreage, (H) fungicide-treated acreage, (I) insecticide-treated acreage, and (J) manure-treated acreage. PSCI, Penn State Cancer Institute; SVI, social vulnerability index; UVR, ultraviolet radiation.

The UVR ranged from 3,701 to 3,988 Wh/m^2^ and followed a south-to-north gradient, with slightly higher averages in south central counties. SVI scores showed limited spatial variability across counties and no consistent clustering pattern (Fig 2; Data Supplement, Tables S1B-S1E).

Agricultural land use demonstrated marked regional differences. The mean proportion of agricultural land per county was 22.1% statewide, compared with 33.2% among south central counties and 18.9% elsewhere. Cultivated land was particularly concentrated in the south central cluster, averaging 19.8% which was nearly triple the value, 6.9%, observed for other counties.

Agrochemically treated acreage mirrored these patterns. Herbicide use was the most prevalent, averaging 16.9% of land per county in south central counties versus 6.5% elsewhere. Although insecticide- and fungicide-treated land accounted for smaller proportions overall, each was more prevalent in south central counties.

### Regression Analysis

#### Covariates UVR and SVI

All models included UVR and SVI to adjust for potential confounding. UVR was not statistically significant in any model although coefficients were negative (incidence rate ratio [IRR], 0.970 and 0.971; Table 1). SVI consistently emerged as a significant inverse predictor of melanoma incidence (IRR, 0.919 and 0.921, respectively, *P* ≤ .002). In sensitivity analyses replacing SVI with SVI-T3, the association shifted positive in each model, reaching significance only in the herbicide model (cultivated IRR, 1.067, *P* = .094; herbicide IRR, 1.081, *P* = .046; Data Supplement, Table S2). Within each SVI specification, adding agricultural exposures improved fit relative to reduced models with covariates alone. Across specifications, models using SVI and SVI-T3 had a very similar fit, with no consistent advantage of either index (Data Supplement, Table S3).

**TABLE 1.** Spatial Adjacency-Weighted COM-Poisson Regression Results for Cultivated Land Use, Herbicide-Treated Acreage, and Melanoma Incidence Among Adults 50 Years and Older in PA Counties (2017-2021)

Model Variable	β (estimate)	IRR (95% CI)	*P*
Reduced model			
UVR (Wh/m^2^)	0.025	1.025 (0.94 to 1.12)	.586
SVI (percentile)	–0.073	0.930 (0.88 to 0.98)	.012
Cultivated land model			
Cultivated land, % of county area	0.132	1.141 (1.07 to 1.22)	<.001
UVR (Wh/m^2^)	–0.030	0.970 (0.89 to 1.05)	.471
SVI (percentile)	–0.084	0.919 (0.87 to 0.97)	.001
Herbicide model			
Herbicide-treated acreage, % of county area	0.126	1.135 (1.06 to 1.21)	<.001
UVR (Wh/m^2^)	–0.029	0.971 (0.89 to 1.06)	.493
SVI (percentile)	–0.082	0.921 (0.88 to 0.97)	.002

NOTE. Herbicide (%) refers to herbicide-treated acreage as a percentage of total county land area. Each 8.6% increase in herbicide-treated acreage was associated with a 13.5% increase in melanoma incidence. Each 10% increase in cultivated land was associated with a 14.1% increase in melanoma incidence. All models were adjusted for UVR and SVI. Model fit statistics: Reduced model: logLik = –315.74, mAIC = 643.47, cAIC = 557.48, df = 14.77, pseudo-R^2^ = 0.1639; cultivated land model: logLik = –308.74, mAIC = 631.49, cAIC = 555.20, df = 17.30, pseudo-R^2^ = 0.1824; and herbicide model: logLik = –309.81, mAIC = 633.62, cAIC = 556.75, df = 17.10, pseudo-R^2^ = 0.1798.

Abbreviations: β, regression coefficient; AIC, Akaike information criterion; cAIC, conditional AIC; COM-Poisson, Conway-Maxwell Poisson; IRR, incidence rate ratio; logLik, log-likelihood; mAIC, marginal AIC; PA, Pennsylvania; SVI, social vulnerability index; UVR, ultraviolet radiation.

#### Agricultural Land-Use Models

Cultivated land had the strongest association with melanoma incidence. In the cultivated land model, each 10.3% increase in cultivated land (one standard deviation [SD]) was associated with a 14% increase in melanoma incidence (IRR, 1.141, *P* < .001; Table 1). In trivariate models, total agricultural land also showed a positive association (IRR, 1.113, *P* = .001), but in multivariable models including both cultivated and total agricultural land, only cultivated land remained statistically significant. Pasture/hay land was not significantly associated with incidence in trivariate models, but demonstrated a marginally statistically significant negative association when included with total agricultural land (IRR, 0.918, *P* = .048). Based on coefficient estimates, model fit, and parsimony, cultivated land was selected as the final land-use variable (Table 2; Data Supplement, Table S4).

**TABLE 2.** Comparative Model Fit Statistics for Spatial COM-Poisson Models Evaluating Land-Use and Agrochemical Variables of Melanoma Incidence

Model	logLik	mAIC	cAIC	edf	Pseudo-R^2^
Reduced model					
UVR + SVI	–315.74	643.47	557.48	14.77	0.164
Land-use models					
Total agricultural land	−310.60	635.19	556.54	16.79	0.178
Cultivated land	−308.74	631.49	555.20	17.30	0.182
Pasture/hay land	−315.20	644.41	557.96	14.88	0.165
Total ag + cultivated	−308.74	633.47	555.63	17.06	0.183
Total ag + pasture/hay	−308.71	633.42	555.62	17.06	0.183
Cultivated + pasture/hay	−308.73	633.47	555.62	17.06	0.183
Agrochemical models					
Herbicide	−309.66	633.32	556.06	17.10	0.180
Fungicide	−310.18	634.37	556.47	17.06	0.179
Insecticide	−311.90	637.81	557.10	16.26	0.174
Manure	−313.51	641.01	557.41	15.58	0.170
Herbicide + fungicide	−309.09	634.18	556.30	17.25	0.182
Insecticide + fungicide	−310.18	636.37	556.74	16.92	0.179
Herbicide + manure	−309.21	634.43	555.92	18.24	0.181
Insecticide + manure	−311.90	639.80	557.39	16.57	0.174
Fungicide + manure	−309.82	635.63	556.71	17.83	0.180

NOTE. All models included UVR exposure and SVI as covariates and were log-linked to population size. Land-use models included total agricultural land, cultivated land, pasture/hay, and their combinations. Agrochemical models included herbicide-, fungicide-, insecticide-, and manure-treated acreage, separately and in combination. Pseudo-R^2^ values represent McFadden's pseudo-R^2^, calculated as 1–(logLik_model/logLik_null), using an intercept-only COM-Poisson null model with a spatial structure. Bold rows indicate the best-fitting models based on AIC and pseudo-R^2^ values.

Abbreviations: AIC, Akaike information criterion; cAIC, conditional AIC; COM-Poisson, Conway-Maxwell Poisson; edf, effective df; logLik, log-likelihood; mAIC, marginal Akaike information criterion; SVI, social vulnerability index; UVR, ultraviolet radiation.

#### Agrochemical Models

Among agrochemical variables, herbicide-treated acreage demonstrated the strongest and most consistent association with incidence. Each 8.6% increase in herbicide-treated acreage (one SD) was associated with a 13.5% increase in melanoma incidence (IRR, 1.135, *P* < .001; Table 1). Positive associations were also observed for fungicide- (IRR, 1.117, *P* < .001), insecticide- (IRR, 1.104, *P* = .004), and manure-treated acreage (IRR, 1.075, *P* = .029) although these estimates were weaker and more sensitive to model specification (Data Supplement, Table S5).

Multivariable models including multiple agrochemical variables were limited by multicollinearity and did not improve fit over the herbicide-only model. In paired models, only herbicide (in the herbicide + manure model; *P* = .002) and fungicide (in the fungicide + manure model; *P* = .004) remained significant. Based on strength, parsimony, and model performance, herbicide-treated acreage was retained as the final agrochemical variable (Tables 1 and 2; Data Supplement, Table S5).

#### Analyses by Cluster Status

To evaluate geospatial heterogeneity in associations, we stratified models by LISA-defined cluster status. The south central high-incidence cluster (n = 15) was compared with all other counties, referred to as the noncluster group (n = 52). Among noncluster counties, both cultivated land (IRR, 1.150, *P* = .001) and herbicide-treated acreage (IRR, 1.159, *P* < .001) were positively associated with melanoma incidence. However, within the high-incidence cluster, these associations, cultivated land (IRR, 1.003, *P* = .927) and herbicide-treated acreage (IRR, 0.949, *P* = .204), were attenuated and no longer significant (Data Supplement, Table S6).

**FIG 3.**
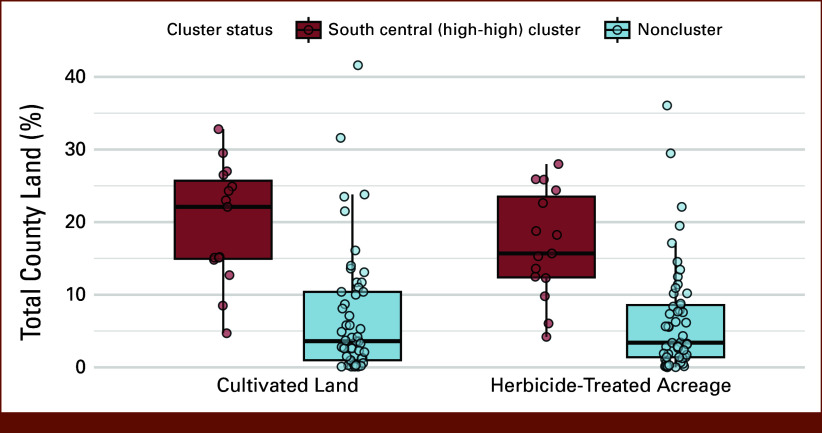
Boxplots of cultivated land and herbicide-treated acreage stratified by LISA cluster status. Cultivated land and herbicide-treated acreage are expressed as percentage of total county land, stratified by spatial cluster status, Southcentral cluster (n = 15) versus noncluster (n = 52). The horizontal line in each boxplot represents the median; circles indicate individual counties. Southcentral cluster counties had significantly higher median values of cultivated land (22.1% *v* 3.6%) and herbicide-treated acreage (15.7% *v* 3.4%) compared with noncluster counties (*P* < .001 for both, Wilcoxon rank-sum test). Mean values were also elevated in cluster counties: cultivated land (mean = 19.8% *v* 6.9%) and herbicide-treated acreage (mean = 16.9% *v* 6.5%). Counties in the Southcentral (high -high) cluster exhibited significantly higher percentages of both cultivated land (*P* < .001) and herbicide-treated acreage (*P* < .001) compared with noncluster counties (Wilcoxon rank-sum test). Spatial clusters were identified using LISA; noncluster counties include all low-low, high-low, and nonsignificant counties. LISA, local indicators of spatial association.

Interaction terms between cluster status and each exposure were not statistically significant (cultivated land × cluster: IRR, 0.923, *P* = .267; herbicide × cluster: IRR, 0.880, *P* = .077). However, south central counties had a significantly higher average percent of cultivated land (19.8% *v* 6.9%, *P* < .001) and herbicide-treated acreage (16.9% *v* 6.5%, *P* < .001) than noncluster counties (Fig 3).

## DISCUSSION

This study identified a 15-county geospatial cluster of elevated melanoma incidence in South Central PA. We also identified significant associations between melanoma incidence and agricultural practices and patterns, independent of ambient UVR and social vulnerability. Specifically, each 10.3% increase in cultivated land was associated with a 14% increase in melanoma incidence, whereas each 8.6% increase in herbicide-treated acreage corresponded to a 13.5% increase in incidence.

Our findings contribute to evidence that agricultural patterns and practices may increase melanoma risk. Similar associations have been reported in agricultural regions of Utah, Poland, and Italy.^52-54^ Higher melanoma rates among farm workers, compared with other outdoor workers, support agriculture-specific exposures.^55^ Importantly, Carozza et al^56^ observed a dose-dependent increase in childhood melanoma in high-cropland counties. Similarly, an Iowa cohort study found elevated melanoma mortality among younger farmers.^57^ In addition to exposure through direct application,^17^ pesticide exposure can also occur to nearby individuals through drift, volatilization, contaminated dust, and runoff.^58^ Elevated pesticide concentrations have been recorded in homes near cropland, reinforcing potential impacts on nonoccupational populations.^59-62^

While herbicides demonstrated the strongest and most consistent association in our study, we also observed positive associations for insecticide-, fungicide-, and manure-treated acreage. These estimates were smaller and more model-sensitive, likely because of lower usage or collinearity with herbicides. Nevertheless, the insecticide and fungicide findings are consistent with previous studies.^8,17,25^

Pasture/hay land was not significantly associated with melanoma incidence, consistent with studies suggesting that grain- and oilseed-related practices may pose greater risk than livestock-related practices.^19^ However, differences in regional practices and occupational roles may influence exposure heterogeneity affecting inconsistencies across studies.^63^

Stratified models showed significant associations between melanoma incidence and each cultivated land and herbicide-treated acreage in noncluster counties, but not within the high-incidence cluster. This attenuation may reflect reduced statistical power (n = 15) or saturation effects in regions where both exposures and melanoma incidence are already elevated. While interaction terms were not statistically significant, geospatial heterogeneity remains plausible.

Although not our primary hypothesis, both SVI and UVR yielded informative insights. SVI was inversely associated with melanoma incidence across all models, consistent with the literature linking higher melanoma rates to more affluent, socially advantaged populations.^64^ In sensitivity analyses, the racial/ethnic minority population (SVI-T3) did not attenuate the effects of agriculture, our primary hypothesis. SVI-T3 was positively associated with incidence in the herbicide model, which is counterintuitive because Hispanic people and Black people have lower incidence of melanoma than do non-Hispanic White people. However, the ecologic design of our study limits inference of individual risks. Importantly, several of the cluster counties have a substantial population of Plain people (eg, Amish, Mennonite) who are a distinct ethnic population and often involved in agriculture.

Despite it being a leading environmental risk, UVR was not a significant predictor in any model. This may be a result of the relatively narrow south-to-north gradient of ambient UVR across PA. By contrast, larger differences in UVR may be more relevant in geographically larger melanoma patterns.

Notably, the high-incidence melanoma cluster in South Central PA lies entirely within PSCI's 28-county catchment area.^31^ This geographic alignment presents a strategic opportunity to integrate PSCI's research, outreach, engagement, and clinical care with the region's elevated cancer burden. As the land-grant university for PA, Penn State University's extensive engagement with the agricultural sector can strengthen PSCI's outreach and engagement initiatives across these high-burden counties, all within its catchment area.

Our findings support the application of a One Health approach to cancer control, which recognizes the interdependence of human, environmental, and agricultural systems.^65^ Lengerich's expanded One Rural Health model extends this systems-based framework to include social determinants of health in rural cancer control strategies. For instance, melanoma prevention in the south central cluster could involve an integrated approach to concurrently address occupational, environmental, and behavioral risks of agricultural workers.

Beyond individual-based interventions, integrated pest management, consistent with the One Rural Health approach, offers a means to reduce agrochemical reliance and potentially mitigate associated melanoma risk.^66-68^ National exposure surveillance platforms, such as CAREX Canada, could be adapted for US use and linked with cancer registries to facilitate early detection of environmental carcinogens.^69^ Community engagement and education on safe pesticide practices remain essential. Notably, in 2016, over one quarter of all agricultural pesticide use in the United States involved chemicals banned in the European Union.^70^

Finally, this study challenges conventional approaches to defining rurality based solely on population density and community patterns. Eight of the 15 high-incidence cluster counties are designated as metropolitan (nonrural),^71^ despite having substantial agricultural activity. As Lengerich has argued,^72^ relying solely on such metrics may misclassify functionally rural communities, obscuring key occupational and environmental risks relevant to cancer control.

As an ecologic study with cross-sectional data, our design does not allow for individual-level (causal) inference or temporal analysis. Melanoma incidence has been found to be under-reported across state registries.^73^ Results may not be generalizable to regions with different agricultural practices, environments, or demographics.

Agrochemical data captured only agricultural applications and lacked details on used patterns, protective behaviors, and direct human exposure. Agricultural exposures likely affect subpopulations in the county, and ambient exposure pathways, such as pesticide drift or runoff, may broaden the population affected. UVR estimates reflected long-term county averages and could not account for variation from individual behavior or susceptibility. Collinearity among exposure variables also constrained multivariable modeling and limited our ability to isolate independent effects. The impact of unmeasured confounders, such as access to care, genetic predisposition, or other environmental exposures, cannot be excluded.

Despite these limitations, the results of this study provide a population-based estimate of melanoma risk from agricultural practices and patterns in PA, with a cluster in South Central PA. As such, they can directly facilitate cancer control research. Future etiologic research could incorporate these results into case-control studies in South Central PA that would explore individual, farm, and environmental risk factors. Inclusion of biomonitoring, such as serum pesticide metabolite levels, could provide individual-level validation of our ecologic association. In addition, future research could confirm and specify outcomes, such as melanoma subtypes. Comparative analyses with other agricultural regions may reveal variations in agricultural risk and increase generalizability. Longitudinal cohorts could clarify temporal relationships. Finally, future investigations could focus on populations including Plain communities and prevention efforts within PSCI's catchment area.

In conclusion, this study identified a high-incidence melanoma cluster in South Central PA and found significant associations between melanoma incidence and both cultivated land and herbicide-treated acreage. Within the cluster, melanoma incidence among adults 50 years and older was 1.57 times the statewide average. To our knowledge, this is the first population-based study to find that specific agricultural patterns and practices may contribute to geospatial disparities in melanoma risk. Importantly, our findings support a One Health approach to reducing the impact of this cancer disparity within a cancer center's catchment area.

## DATA SHARING STATEMENT

A data sharing statement provided by the authors is available with this article at DOI https://doi.org/10.1200/CCI-25-00160.
